
JOURNAL INFORMATION
==============================
NLM Title Abbreviation: Med Chir Rev
NLM Journal ID: 0034617

The Medico-Chirurgical Review

Publisher: Biomedical Journal Digitization Project

ARTICLE INFORMATION
==============================
PMCID: PMC4966186
PMID: 29917608
Article version: 1
Subjects: Reviews

Dr. Harrison on Spinal Diseases

Print publication date: 1828 Jul 1
Volume: 9
Issue: 17
First page: 87
Last page: 90
1828 May 24
License: This work is free of known copyright restrictions. For more information, please see PMC Back Issue Digitization.
License URL: https://creativecommons.org/publicdomain/mark/1.0/

==============================

### IX.

*Pathological and Practical Observations on Spinal Diseases; illustrated with Cases and Engravings. Also, an Inquiry into the Origin and Cure of Distorted Limbs.* By Edward Harrison, M.D. F.R.A.S. Ed. Royal 8vo. pp. 294, with 15 plates. Price One Guinea. T. & G. Underwood, 1827.

There are few men who have had more opportunities of studying spinal distortions and diseases than Dr. Harrison—and, therefore, if he has profited by his experience, and laid the results of his observations candidly before the public, his book must prove valuable to society at large. Dr. H. assures us that spinal distortions, from constitutional causes, are become so universally prevalent, "that we meet with but few females, above the condition of laborious servitude, whose backs are perfectly straight." If this be true—and we fear it is not very far from the truth—then the progress of civilization and the "march of intellect," like the "paths of glory," "lead but to the grave." We say the grave—for were spinal distortions only productive of deformity, no great evil would result. The pride of the young belle might be lowered, and the labours of the stay-maker might be increased—and there the mischief would end. But, alas! the unsightliness is the smallest part of the misfortune.

"A little consideration will be sufficient to shew that the spine constitutes an essential portion of many animals, and that in the human body a greater variety of complicated sufferings arise out of its derangements than from those of any other organ. It affords support to the head, ribs, arms, and internal viscera. Numerous nerves issue out of its cavity, to be distributed over the skull, trunk, and limbs. Others strike inwards to the internal structures, and regulate their vital functions. It follows that the disorder of an organ, which directs the motions of so many members, and is besides destined to control such numerous, complicated, and diversified operations, must be capable of producing the most distressing effects."

Dr. Harrison does not decide whether these maladies have lately increased, as is generally supposed—but he has little hesitation in asserting that, "in our cold and variable climate, few delicate girls are wholly exempt from their influence." Whether the distortion be a lateral or spiral curvature—a projection outwards, constituting the hump-back or curved spine of Pott—the anterior or forward curve—displacements of single vertebræ—or, which is more common, a deformity compounded of some or all of these varieties, Dr. H. maintains but one kind of pathology for all.

"Viewing matters in this light, I have ventured to maintain, that *where the organisation remains sound* the several examples of spinal distortion are occasioned by the ligaments giving way irregularly, and suffering the vertebræ to slide from their proper stations in the column.

"Whatever occasions the ligaments to elongate, may, at the same time, permit them to stretch unequally. Where this happens, the joints will be likely to be protruded in that direction, because they will there encounter the least resistance.

"It is to this simple principle alone, as I conceive, that the various constitutional distortions owe their form. If the protrusion be backward, a gibbous swelling or hump back will follow. The curve is anterior or forward when the spinal column sinks inwards. The sigmoidal, lateral, or serpentine deflection, is occasioned by the ligaments allowing the vertebræ to make a deviation generally towards the right, between the shoulders, and another towards the left, in the loins."

There is some ambiguity in the above passage; for surely, if, from constitutional disorder, (to which Dr. H. traces these distortions) the ligaments are so weakened, as to "give way irregularly," their organization cannot be called sound. If the coats of an artery give way irregularly, so as to form false aneurism, can its organization be considered sound?

Dr. H. informs us, that his researches have been almost entirely confined to that species of spinal complaints which originates in constitutional debility. He does not extend his inquiries to the varieties proceeding from hurts, the destruction of parts, and other causes. His view of spinal complaints being (he thinks) in several respects new, he determined to confine his observations, in the first instance, to the faculty exclusively—and, therefore, almost the whole of the present expensive volume was published, in a series of essays, in the Medical and Physical Journal. But having had wider experience, and (which is rather remarkable) "more time for reflection," he now "ventures upon a BOLDER FLIGHT, by addressing himself to the GENERAL READER." Now, in our humble opinion, this "bolder flight" is like that of ICARUS, a complete descent. Any dunce—any quack, may address himself, and with very considerable success, to the GENERAL READER;—but when he soars, on Icarian wings, to the professional public, the wax dissolves—and down he goes! The foregoing declaration is the most injudicious that Dr. Harrison could have possibly made. He tells us that it is now more than eight years since he first *imbibed* his present opinions respecting spinal pathology. We find that the essays composing the volume before us began to be published eight years ago—and are now merely republished, with the addition of plates. The inevitable conclusion is, that these essays were essays tentative—that is, they were a series of special pleadings, unsupported by the only sure basis, morbid anatomy—and probably, at that time, grounded on very few personal observations. This consideration greatly lessens our confidence in Dr. Harrison's doctrines. Indeed the want of morbid anatomy, and the exuberance of unsupported and gratuitous assertion, are fatal to this volume. What must the anatomist and physiologist think of such arguments as the following in support of *ligamentous*, and in contradiction of *muscular* debility as the cause of spinal distortions?

"Were spinal distortions to originate in the muscles, as some experienced practitioners affirm, we should find them most prevalent among the agricultural youth of both sexes, who, in carrying heavy burdens, and performing other laborious works, bring their muscles into the most vigorous and long-continued contractions; yet it is well known that the disorder seldom appears among them, and, when it does shew itself, can be traced to some obvious cause. It follows from this exemption among our brawny labourers, that spinal maladies are seldom produced by muscular action alone, unless where existing infirmity in the vertebral column favours its operation."

Who ever dreamt or said that active muscular action of the spine could possibly conduce to spinal distortions? Did the late Mr. Wilson, the late Mr. Shaw, or the late Mr. Bampfield ever preach such doctrines? No! they taught the very reverse—namely, that want of exercise weakened the spinal, as it did all other muscles, in consequence of which the spinal column was not properly supported, and curvatures took place. Did not Mr. Wilson recommend a weight to be carried on the head, for the specific purpose of exercising and strengthening the spinal muscles? How then could Dr. Harrison bring forward such *distorted* representations of other mens' sentiments?

And what proofs, from anatomy, physiology, or analogy, does Dr. H. adduce to shew that the ligaments, rather than the muscles of the spine, are weakened by constitutional disorder? Not one that is, in the slightest degree, satisfactory. We see the muscular system immediately prostrated by any constitutional indisposition—and that in a far more rapid manner, and to a far greater extent, than ligaments and tendons. Yet to maintain a new or peculiar doctrine, we must be forced to believe that it is on the ligaments of the spine, rather than on the muscles, that constitutional disorders exert their debilitating influence. Speaking of exercise as a mean of strengthening the spinal muscles and lessening spinal curvature, Dr. H. gives us the following assertions instead of proofs.

"Has the vertebral column been restored, in a single instance, to its natural shape during this treatment? Has the cure been rendered more perfect, or been finished in less time, with these auxiliaries, than by adopting the horizontal position and resting contrivances of Mr. Baynton? According to my observations, the result has always been unfavourable, both in time and all other respects. Nor should this excite surprise: the disorder is situated in the articulating structure below the muscles; their motions, at every turn and change, necessarily agitate and disturb the vertebral joints, through their tendinous attachments, and the cure is impeded, rendered less certain, and less perfect, than if the treatment had been restricted to rest alone.

"Highly as I approve of Mr. Baynton's plan, in taking off the incumbent weight, in giving the muscles and tendinous fibres an opportunity to adapt themselves to their new situations, I think it is extremely defective, *because it encourages no trials to replace the luxated vertebræ in their former positions.* It, therefore, leaves the patient in a state of deformity, and, as an unavoidable consequence of it, in bad health."

"*Happily, these vertebral dislocations, from internal causes, may be easily removed in every recent, and in many old cases,* so as to leave no traces in the appearance of the back, or in the health of the individual."

In the above passage, the whole jet of the new doctrine is, intentionally or unintentionally, disclosed—at least to the professional reader. Every case of distorted spine depends on relaxation of certain ligaments, and partial luxation of certain vertebræ—hence, each case differs from another, and requires the discriminating eye and adroit manipulation of the practitioner. God forbid that we should impute to Dr. Harrison, or to any other doctor, the remotest idea of charlatannerie; but we do believe that it would be difficult to devise a system better adapted to the practice of that art, than the spinal pathology here delineated. The following extract of a letter from Dr. Harrison, to a young lady 26 years of age, explanatory of her own case, and his peculiar doctrines, is too delicate a morçeau not to be recorded in the noble *art and mystery* of medicine!

"To me it appears much more probable, that the malady *is wholly confined to the connecting ligaments of the vertebræ.* These having lost their proper tone and firmness, gradually stretched, and grew longer. Owing to this infirmity, the prominent vertebræ separated, and removed further asunder. The elongation would of course lead to some changes in the arrangement of the vertebræ. Being under less confinement, they gradually deserted their former places in the column, to enter into new ones. This alteration of situation is, I think, easily explained upon anatomical principles. None of the vertebræ are in actual contact, or touch, though they approach very nearly to one another in several places. Wheresoever they approximate, something is interposed to prevent their junction and cohesion. The bodies are separated by cartilage, and their processes by a *slimy bag,* which keeps them apart. The ligaments constitute the chief connecting medium of the column. They are naturally firm, strong, and curiously interwoven among the vertebral prominences. So long as they preserve their *strength unimpaired, no change in position,*

*or derangement of parts, can possibly occur.* There is no vacillation, no permanent alteration of form. The *flexions* and *flexures* are performed with ease. These motions having ceased, and the column being again at rest, the parts return to their former places. When, however, a particular attitude is frequently repeated, or long persevered in, the ligaments concerned gradually accommodate themselves to the acquired position. Should this constrained habit be much longer continued, deformity will assuredly supervene. Such is, I conceive, the origin and progress of spinal complaints, when they proceed from weakness in the ligaments. In your case the disorder seems to have commenced in them,' and can, I think, be removed by restoring the extruded vertebræ to their former stations. With a view to effect this desirable change, I propose to employ strong frictions to the prominent vertebræ. These to be followed by well regulated pressure, directed to the same parts. —After the process has concluded, I should confine the vertebræ with long slips of adhesive plaster, to prevent their receding. Lastly, the trunk is to be immediately replaced in the supine direction, and to be furnished with a mechanical contrivance to maintain constant pressure upon the prominent vertebræ. These are some of the means, which I venture to suggest, for the cure of your malady. As the vertebræ project almost straight backwards, I think the proposed plan is more likely to succeed, than if they had taken a lateral, or forward direction. I think it only candid in me to inform you, that after a diligent search among the medical books in my possession, I can no where discover any authority either in support of the doctrine, or the practice, that I have ventured to advocate."

After this specimen of the "bolder flight" which Dr. Harrison has taken from the low ranks of his brethren, to the GENERAL READER, we must lay down the pen. We are positively ashamed (if it will be believed that a REVIEWER can have any sense of shame) to record such a passage from the writings of a physician of the present period, on our pages. But the above extract will characterise the book under review, better than any thing which we could say. The pathology is erroneous—the practice is, for the most part, concealed—and the plates are more calculated to frighten the GENERAL READER than to convey any information to the MEDICAL PRACTITIONER.

---

### X.

*Hints to young Medical Officers of the Army on the Examination of Recruits, and respecting the feigned Disabilities of Soldiers; with Official Documents, and the Regulations for the Inspection of Conscripts for the French and Prussian Armies.* By HENRY MARSHALL, Surgeon to the Forces. Octavo, 224. London, 1828.

THAT portion of Forensic or Judicial Medicine which more particularly belongs to the military service of the country, we believe, has not hitherto been treated, by any person, in a distinct work. We are aware that the subject has been incidentally touched by several authors of the highest repute, viz. Dr. Gordon Smith, Messrs. Paris and Fonblanque, Dr. Beck, and particularly Dr. Hennen, in his "Principles of Military Surgery." But, it has long been a desirable object that the military surgeon should be put into possession of a portable work, to serve him as a manual, upon the feigned defects and disabilities of recruits and soldiers, apart from the extensive range of medical jurisprudence, taken in the valuable works above-mentioned, and which, from their very nature, are too voluminous for the library of a military medical officer.
